# Protein aggregation profile of the human kinome

**DOI:** 10.3389/fphys.2012.00438

**Published:** 2012-11-20

**Authors:** Ricardo Graña-Montes, Ricardo Sant'Anna de Oliveira, Salvador Ventura

**Affiliations:** ^1^Institut de Biotecnologia i de Biomedicina and Departament de Bioquímica i Biologia Molecular, Universitat Autònoma de BarcelonaBellaterra (Barcelona), Spain; ^2^Instituto de Bioquímica Médica, Universidade Federal do Rio de JaneiroRio de Janeiro, Brazil

**Keywords:** protein kinases, protein aggregation, amyloid, protein evolution, AGGRESCAN

## Abstract

Protein aggregation into amyloid fibrils is associated with the onset of an increasing number of human disorders, including Alzheimer's disease, diabetes, and some types of cancer. The ability to form toxic amyloids appears to be a property of most polypeptides. Accordingly, it has been proposed that reducing aggregation and its effect in cell fitness is a driving force in the evolution of proteins sequences. This control of protein solubility should be especially important for regulatory hubs in biological networks, like protein kinases. These enzymes are implicated in practically all processes in normal and abnormal cell physiology, and phosphorylation is one of the most frequent protein modifications used to control protein activity. Here, we use the AGGRESCAN algorithm to study the aggregation propensity of kinase sequences. We compared them with the rest of globular proteins to decipher whether they display differential aggregation properties. In addition, we compared the human kinase complement with the kinomes of other organisms to see if we can identify any evolutionary trend in the aggregational properties of this protein superfamily. Our analysis indicates that kinase domains display significant aggregation propensity, a property that decreases with increasing organism complexity.

## Introduction

Most polypeptides need to fold into specific three-dimensional structures to perform their biological functions (Dobson, 2003). Only the correctly folded forms of these proteins remain soluble in the cell and are able to interact with their molecular targets (Daggett and Fersht, 2009). Therefore, protein misfolding impairs cell fitness and is being found linked to an increasing number of human degenerative diseases. In these disorders, misfolded conformers establish non-native intermolecular contacts that result in their deposition into insoluble amyloid aggregates in the intra- or extracellular space (Chiti and Dobson, 2006). All these assemblies display a common cross-β motif (Nelson and Eisenberg, 2006). However, the ability to form amyloid-like structures is not restricted to a subset of disease-linked proteins and this conformation may be accessed by most, if not all, proteins in living organisms, from bacteria to human, irrespective of their native fold (Dobson, 2004; Jahn and Radford, 2005; de Groot et al., 2009). In fact, the molecular interactions leading to the formation of amyloids are similar to those promoting the folding and functional assembly of proteins (Linding et al., 2004; Castillo and Ventura, 2009). As a result, folding and aggregation pathways are continuously competing in the cell.

Globular proteins are soluble in their biological environments. However, when their stability is compromised by genetic mutations or environmental conditions, local unfolding might promote the exposure to the solvent of aggregation-prone regions previously protected in the native state (Ventura et al., 2004; Ivanova et al., 2006). To confront this danger, protein sequences have evolved strategies to reduce aggregation propensity (Rousseau et al., 2006b; Monsellier and Chiti, 2007; Tartaglia et al., 2007; Castillo et al., 2011). The selective pressure against protein aggregation is stronger for proteins involved in essential cellular functions (Tartaglia and Caflisch, 2007; Chen and Dokholyan, 2008; de Groot and Ventura, 2010), as it might be the case of kinases. Nevertheless, even those globular proteins selected to be highly soluble cannot avoid the presence of aggregation “sensitive” stretches in their sequences (Sabate et al., 2012). Accordingly, we have recently shown that a cancer associated point mutant of human nucleoside diphosphate kinase A displaying reduced conformational stability forms amyloid fibrils under close to physiological conditions (Georgescauld et al., 2011). Similarly, a partially folded intermediate of phosphoglycerate kinase has been shown to self-assemble into amyloid fibrils (Damaschun et al., 2000; Agocs et al., 2010).

The aggregation propensities of proteins are determined to a large extent by their sequences and the intrinsic properties that govern the aggregation of proteins have been already identified. This has allowed the development of a set of algorithms able to predict aggregation-prone regions in protein sequences as well as the overall aggregation propensity of polypeptides (Castillo et al., 2010; Hamodrakas, 2011). Several of these programs are well suited for the analysis of large protein sets, among them AGGRESCAN, an algorithm previously developed by our group (Conchillo-Sole et al., 2007; de Groot et al., 2012), which displays a high power to predict *in vivo* protein aggregation (Belli et al., 2011). Different predictive algorithms have been used to analyze the overall aggregation properties of complete proteomes, from bacteria to human (Tartaglia et al., 2005; Rousseau et al., 2006b; Monsellier et al., 2008; de Groot and Ventura, 2010). Here we address the intrinsic aggregational properties of protein sequences belonging to the same super-family in different organisms. In this way, we have analyzed the aggregation propensities of the protein kinase complements (“kinomes”) of budding yeast, fly, mouse, and humans using AGGRESCAN.

## Results

### Aggregation properties of kinomes

**Figure 1:**
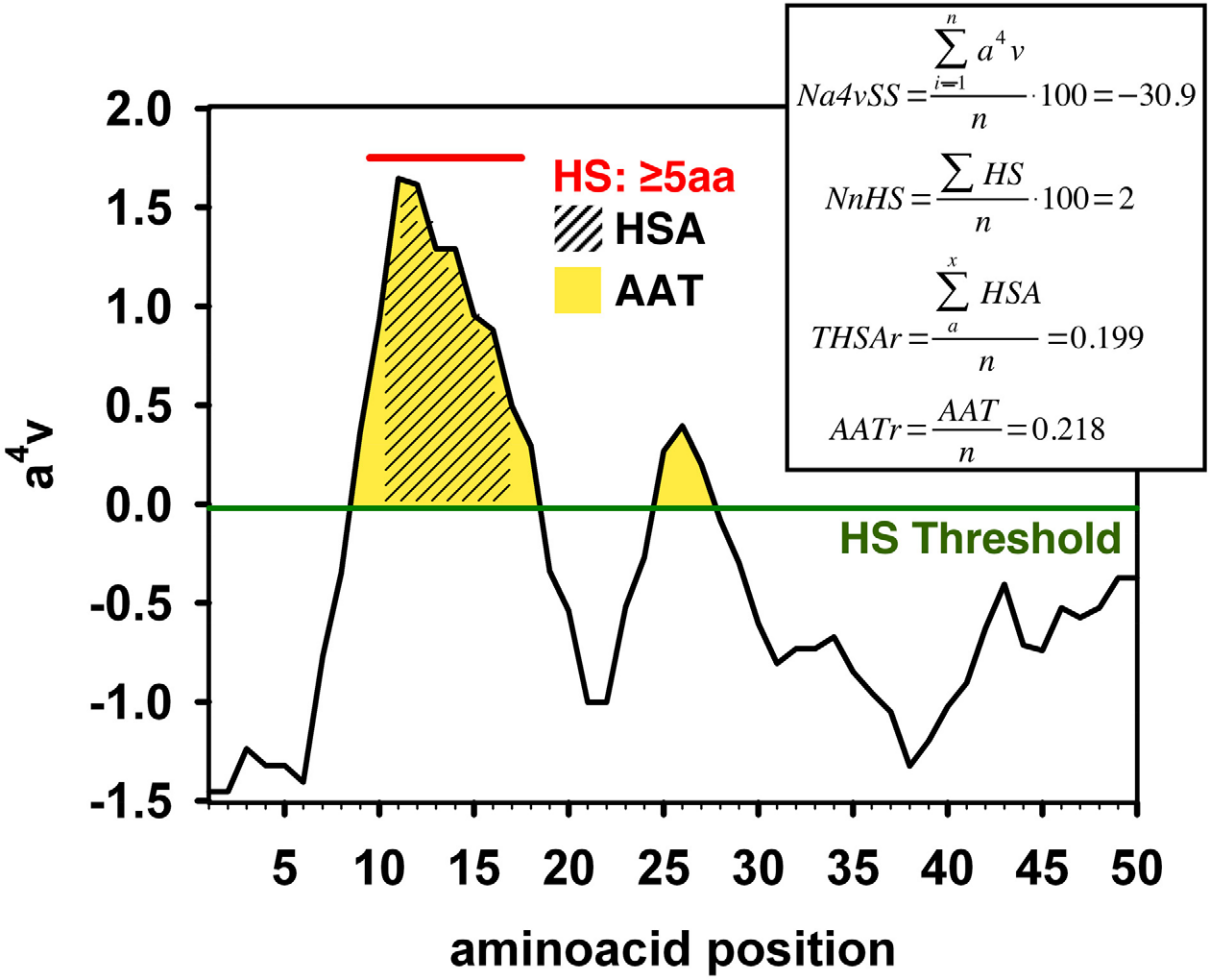
**Example of the AGGRESCAN output with the different parameters calculated by this algorithm**. The aggregation profile is represented as the value of the experimentally derived parameter a^4^v (de Groot et al., 2005) plotted against the query sequence. An overall value of this parameter is given for the whole sequence by dividing the a4v sum for all positions over the number of amino acids an multiplying by 100 (Na4vSS). An aggregation-prone region (APR) or Hot-Spot (HS) is detected whenever a stretch of 5 or more consecutive amino acids, none of them being a proline, with a4v values above the HS Threshold (HST) is found. The normalized number of Hot Spots (NnHS) is calculated as the number of Hot-Spots detected divided by the sequence length and multiplied by 100. The area of the profile above the threshold gives an idea of the aggregation potential of the sequence or a certain region; it can be calculated for the whole profile (AAT) or limiting it to the detected Hot-Spots (HSA). The area values are normalized dividing by the number of amino acids in the input sequence.

We computed the aggregation properties of the complete kinomes of *S. cerevisiae* (104 proteins, all containing a single kinase domain), *D. melanogaster* (197 domains in 194 proteins), *M. musculus* (520 domains in 511 proteins), and *H. Sapiens* (508 domains belonging to 497 proteins) using AGGRESCAN. The following parameters were calculated (Figure 1 and “Materials and Methods”):

1. The average aggregation propensity of the sequence (Na4vSS).
2. The frequency of occurrence of aggregation-prone regions (APR), i.e., the number of aggregating peaks for each 100 protein residues (NnHS).
3. The average aggregating potency of the detected aggregation peaks (THSAr), i.e., the area of the peaks that lies above the detection threshold, normalized by the protein length.
4. The average aggregating potency of residues above the detection threshold (AATr), independently if they are clustered in aggregating peaks or not, i.e., the area of the surface above the detection threshold.

**Figure 2:**
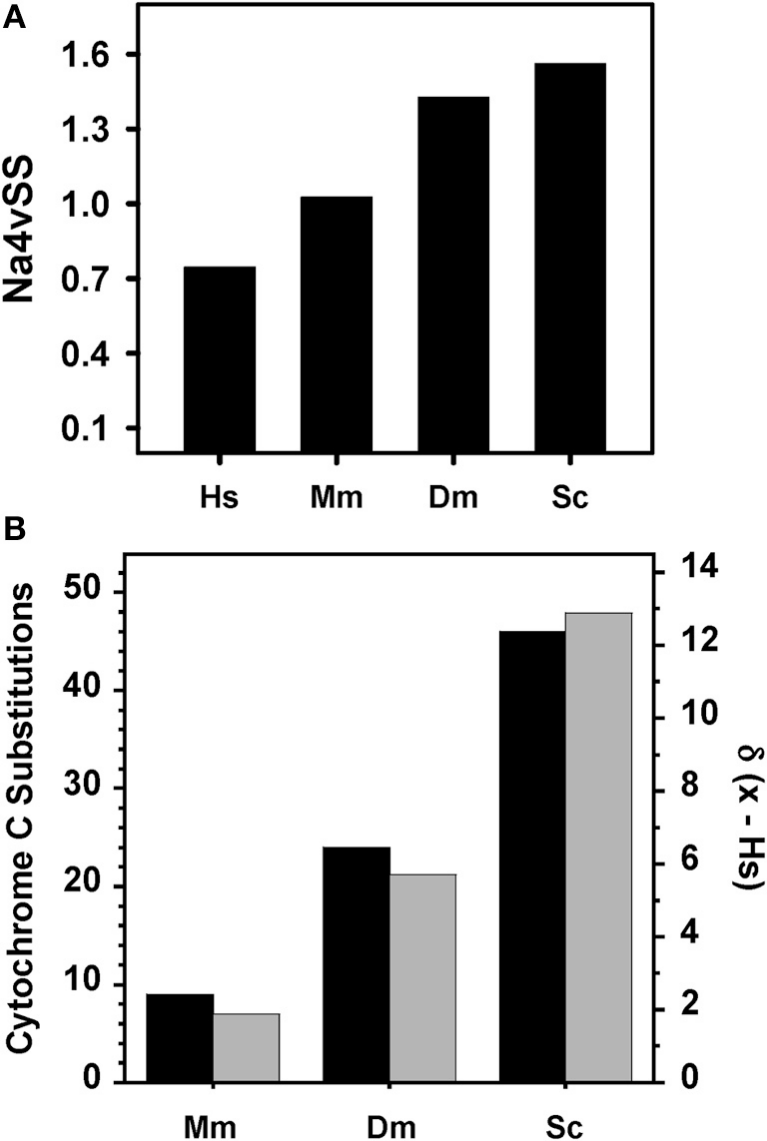
**Relationship between organism complexity and aggregation properties of kinase domains**. **(A)** Average aggregation propensity (Na4vSS) for the complete dataset of kinome domains of human (*Hs*), mouse (*Mm*), fruit fly (*Dm*), and budding yeast (*Sc*). **(B)** Deviation in the distribution of aggregation prone domains (Na4vSS > 0) between human and the rest of species (gray, calculated according to Equation 1) compared with the number of amino acid differences in cytochrome c between human and other species (black).

We retrieved the sequences corresponding to kinase domains in the full-length proteins for the different kinomes and analyzed their aggregation properties. Surprisingly, the calculated average aggregation propensity Na4vSS was positive in the AGGRESCAN scale in all cases, which suggests a certain intrinsic propensity to aggregate for these domain sequences (Figure 2A). Na4vSS of 1.56, 1.42, 1.03, and 0.75 were calculated for yeast, fly, mouse, and human kinomes, respectively. Na4vSS values reflect the average propensity of all the proteins in a dataset. To compare the distribution of domains displaying positive aggregation propensity in the different species, relative to proteins in the Swiss-Prot database, we binned Na4vSS values into 100 groups and calculated the deviation between the human and the rest of kinomes for bins in which Na4vSS > 0.

(1) $$d_{\left(x\;-\text{ human}\right)}=\sum_{i=1}^{N}F\left(Na4vSS_{x}\right)-F\left(Na4vSS_{\text{human}}\right)$$

where *F(Na4vSS_*x*_)* corresponds to the frequency of this bin in the organism *x* and *F(Na4vSS*_human_) is its frequency in the human kinome.

The calculated deviations match well with the evolutive distances in the phylogenetic tree of cytochrome c (Dayhoff et al., 1972) (Figure 2B). Therefore, for kinase domains, it appears that aggregation propensity decreases as we ascend in the evolutionary scale.

**Figure 3:**
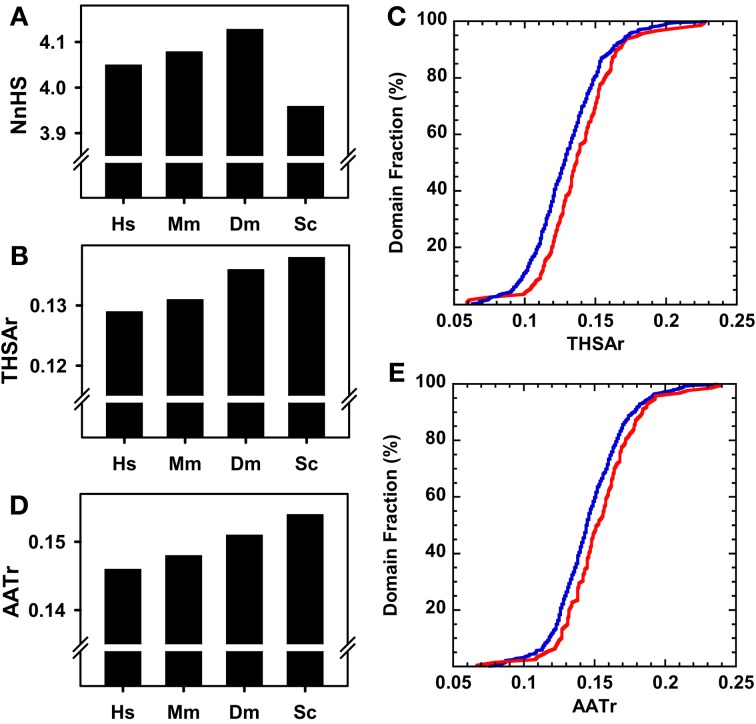
**Aggregation properties of complete kinase domain datasets of different organisms**. **(A)** Normalized number of Hot-spots (NnHS). **(B)** Total Hot-spot area per residue (THSAr). **(C)** Distribution of the THSAr value along the whole dataset. **(D)** Area of the aggregation profile above the Hot-Spot Threshold per Residue (AATr). **(E)** Distribution of the AATr value over the complete kinase domain dataset. In **(A)**, **(B)**, and **(D)**: human (*Hs*), mouse (*Mm*), fruit fly (*Dm*), and budding yeast (*Sc*). In **(C)** and **(E)**: the blue line corresponds to the human dataset and the red line to the yeast kinome.

We explored the reasons for the different aggregation propensities observed in the kinase domains of different species. The frequency of aggregating peaks NnHS is approximately four in all species (Figure 3A). This value is lower in yeast than in humans and therefore it cannot account for the observed differences in overall aggregation propensity. In contrast, the THSAr values follow the trend observed for Na4vSS, indicating that despite sharing similar number of aggregating peaks, the aggregation potency of these regions decreases with organism complexity (Figure 3B). This became more obvious when we compared the cumulative THSAr frequencies in distant organisms, yeast and human (Figure 3C). The 25% of the human kinase domains have a low THSAr (<0.1) in contrast to 5% of yeast domains. On the contrary, 20% of yeast domains display a high THSAr value (>0.15) while only 10% of human domains are included in this set. A similar, trend is observed for AATr values, yet another measure of the aggregation propensity of the sequence (Figures 3D,E).

**Figure 4:**
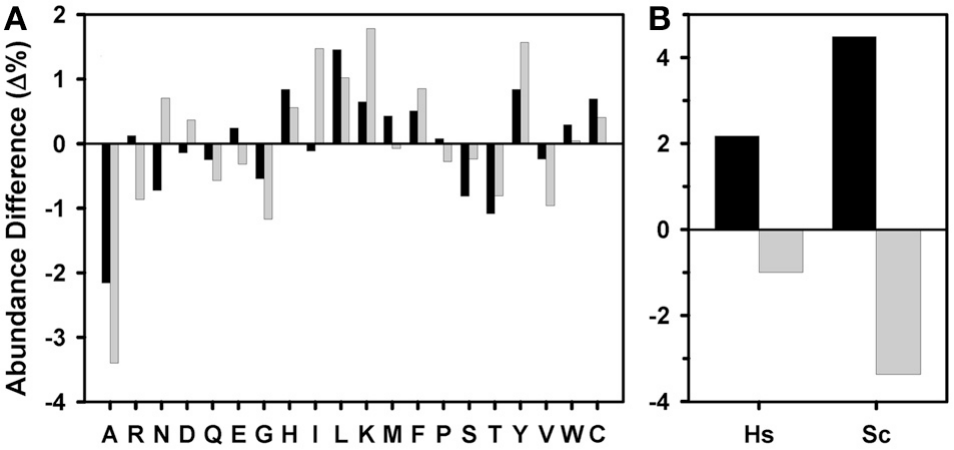
**Amino acid composition of kinase domains**. **(A)** Differential amino acid composition of the human (black) and yeast (gray) kinase domains and **(B)** differential proportion of residues with high (black) and low (gray) β-aggregation propensity in human (Hs) and yeast (Sc) kinase domains, both relative to the amino acid composition of the Swiss-Prot database ensemble (release 2012_07).

To see whether the differences in aggregation propensity of human and yeast sequences might result from an amino acid compositional bias in these species, we compared the amino acid content of yeast and human kinase domains with the average composition of the proteins deposited in Swiss-Prot (Figure 4A). Following the trend described above, both human and yeast kinase domains are, on the average, enriched in residues with high β-aggregation propensity (C, F, I, L, N, Q, V, and Y) and depleted in residues with low β-aggregation propensity (A, G, H, K, P, and R) (Tartaglia et al., 2005). However, these trends are more evident in yeast domains (Figure 4B), in agreement with their overall higher predicted aggregation properties.

### Aggregation properties of the human kinome

**Figure 5:**
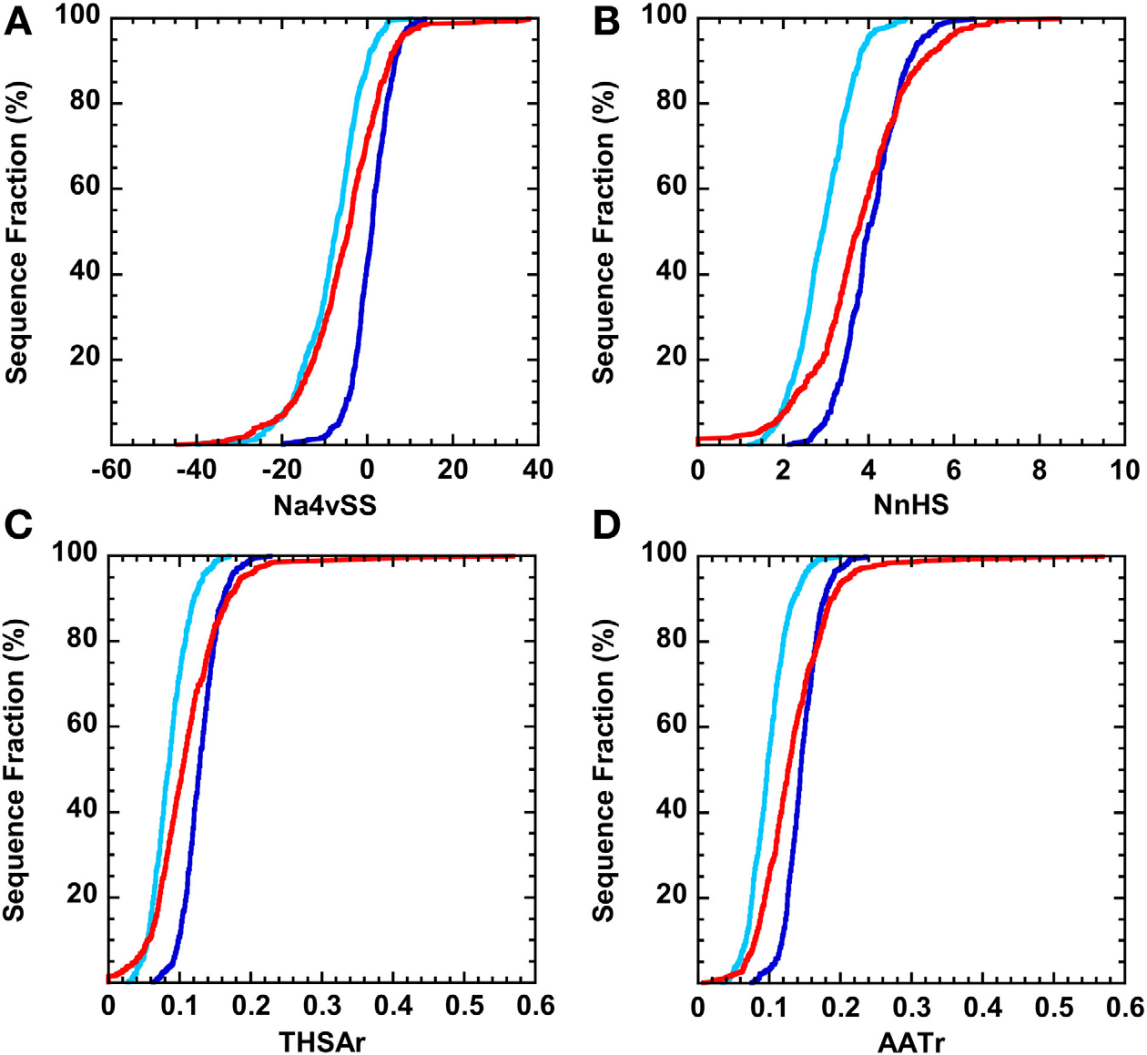
**Distribution of aggregation properties over the complete datasets of human kinase proteins (light blue) and domains (dark blue)**. The red line corresponds to the distribution for a randomized selection of 500 sequences belonging to the Astral40 dataset. **(A)** Average aggregation propensity (Na4vSS). **(B)** Normalized number of Hot-spots (NnHS). **(C)** Total Hot-spot area per residue (THSAr). **(D)** Area of the aggregation profile above the Hot-Spot Threshold per Residue (AATr).

We addressed how the aggregation properties of complete human kinase proteins and their domains compare to those of folded proteins. We used the SCOP-derived database ASTRAL40 (Chandonia et al., 2004) and randomly selected 500 sequences in order to obtain a database similar in size to the human kinome (508 domains and 497 proteins). Proteins in the ASTRAL40 database display higher Na4vSS values than the full-length human kinase protein set (Figure 5A). In addition, on the average, the frequency of aggregation-promoting regions is lower in human kinases than in the ASTRAL40 dataset (Figure 5B). Morevover, kinases tend to have less effective aggregating regions than the selected set of human folded proteins (Figures 5C,D). If we only consider the kinase domains in these proteins we observe the opposite trend. The 60% of kinase domains display positive Na4vSS, in contrast to the 22% of proteins in the ASTRAL40 dataset. In addition, only 2% of kinase domains have high-predicted solubility (Na4vSS < −10), whereas the 30% of folded human proteins display this property.

**Figure 6:**
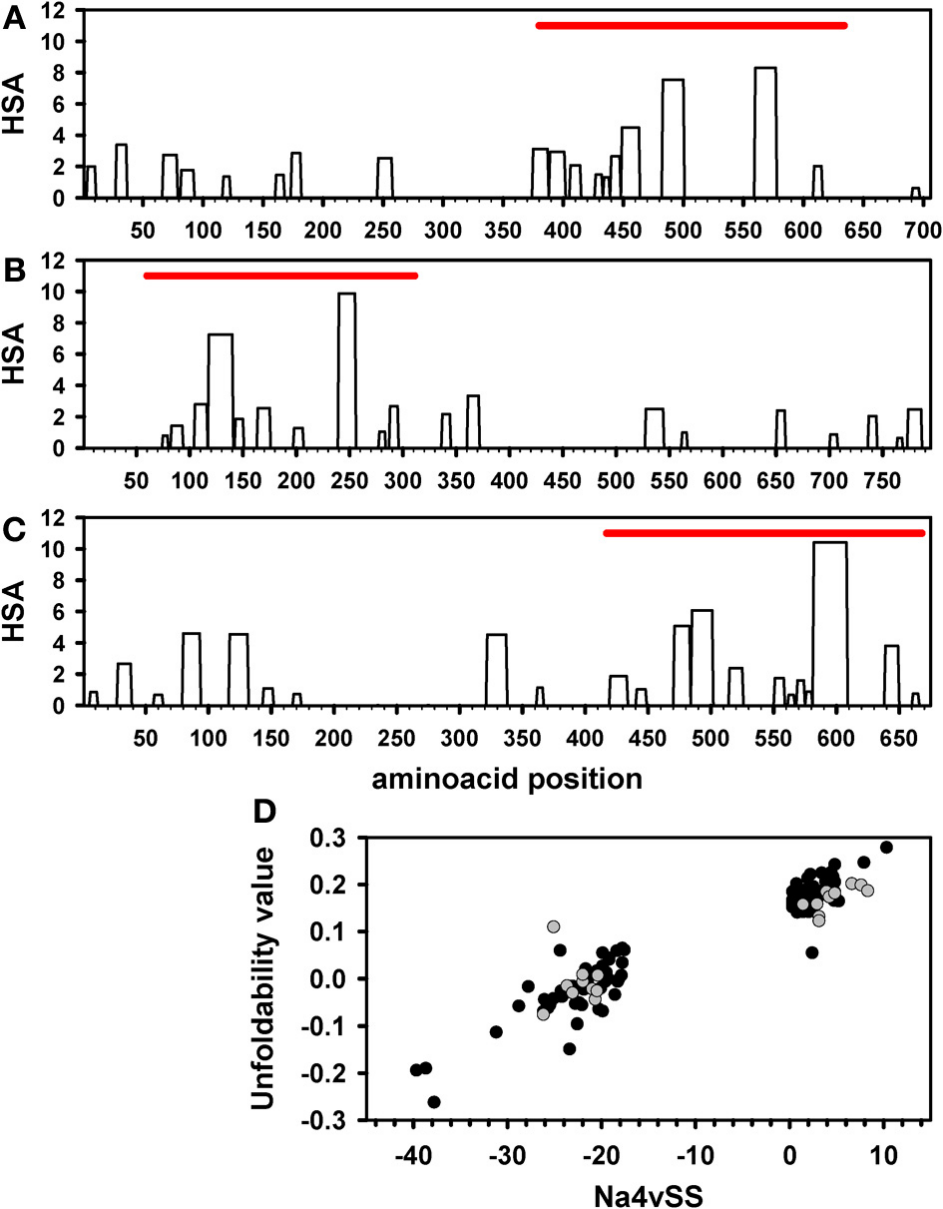
**Aggregation prone-regions and intrinsic disorder in kinases**. AGGRESCAN Hot-Spot Area (HSA) profile for individual kinase proteins: **(A)** PKCt (AGC), **(B)** MARK1 (CAMK), and **(C)** BMX (TK). The position of the corresponding kinase domain is represented by a red bar. **(D)** Intrinsical unfolding propensity of the 10% kinase proteins with the lowest and highest Na4vSS values in the human (black) and yeast (gray) datasets. Intrinsical unfolding propensity predictions were performed using the FoldIndex algorithm.

When we compare human kinase domains with the correspondent full-length proteins, their aggregation properties appear to be strikingly different. For all the parameters, their cumulative frequencies run parallel but always with higher values for the domains alone (Figures 5A–D). This arises from a higher density of aggregating peaks in the domains, displaying also higher potency, compared to the complete protein in which they reside, as shown in Figures 6A–C. It has been shown that intrinsically unstructured proteins (IUPs) and segments are inherently less aggregation-prone than globular proteins (de Groot and Ventura, 2010). Therefore, we wondered if there is any relationship between Na4vSS values and the presence of disordered regions in full-length human proteins. With this aim we calculated the intrinsic unfolding propensity of the 10% protein kinases with the lowest and highest associated Na4vSS values, respectively, using the FoldIndex algorithm. Proteins displaying low intrinsic aggregation propensity are enriched in disordered regions (Figure 6D). This behavior is common to all kinomes, as illustrated in Figure 6D where we compare human and yeast proteins.

### Amyloidogenic regions in human kinase domains

AGGRESCAN is a good predictor of intracellular aggregation propensity and of the regions driving this process; however it is not aimed to identify regions that self-assemble into ordered amyloids, albeit aggregation-prone and amyloidogenic regions coincide in many cases (Rousseau et al., 2006a). To extend our analysis, we have also evaluated the presence of patterns corresponding to hexa-peptides that form amyloid-like fibrils as deduced from experimental studies by de la Paz and Serrano (de la Paz and Serrano, 2004). The patterns were detected using ScanProsite (http://prosite.expasy.org/scanprosite/). One thousand and twenty-nine hits were obtained in 435 out of the 508 active domain sequences that include the human kinome dataset, thus indicating that the presence of sequences able to form amyloid structures, if exposed to solvent, is frequent in kinase domains, many of the sequences containing more than one amyloidogenic stretch.

### Aggregation and amyloidogenic properties of human kinase groups

**Figure 7:**
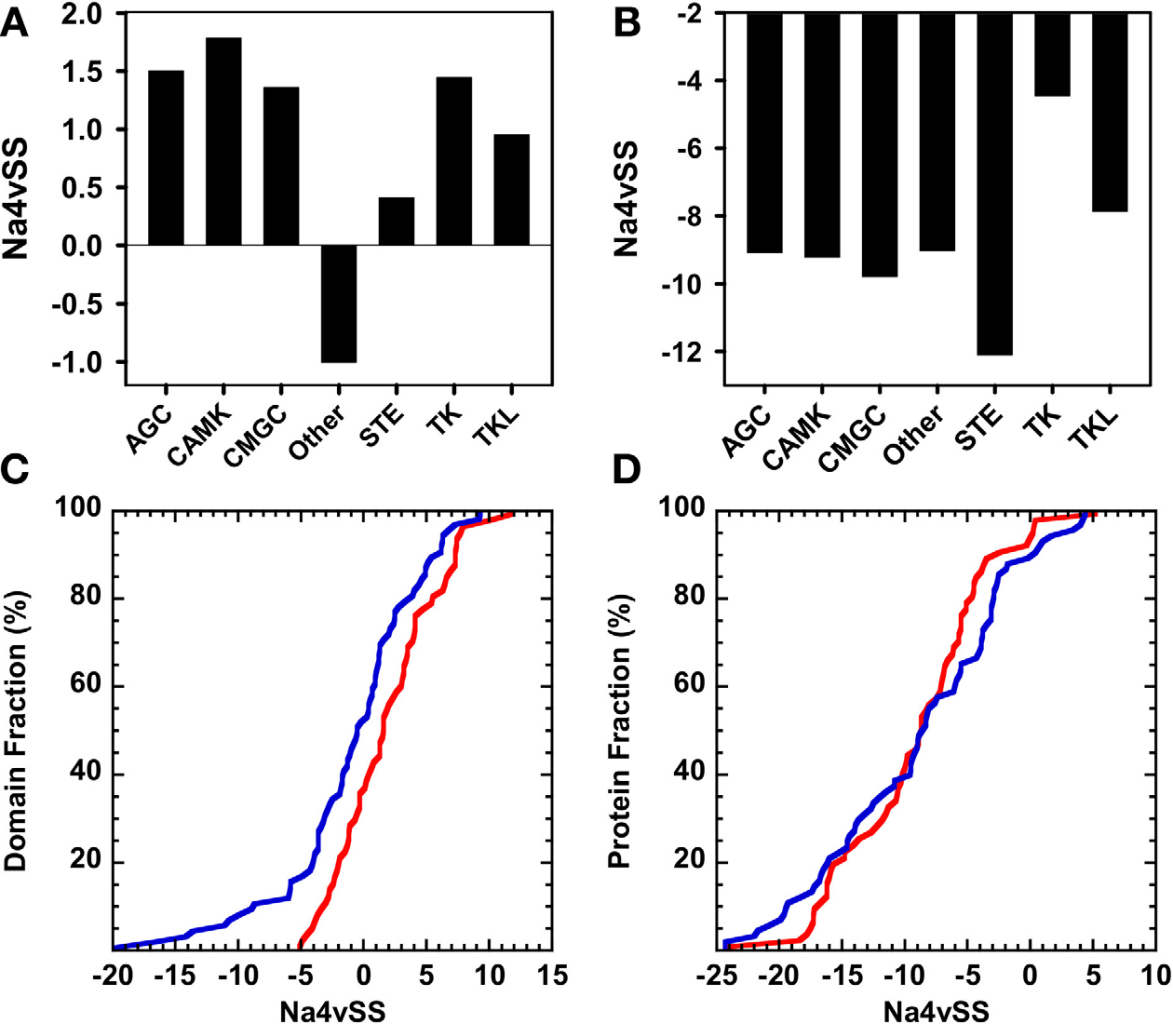
**Aggregation properties of the human kinase proteins and domains clustered according to their corresponding group (only groups composed of >40 sequences are considered)**. **(A)** and **(B)** Average aggregation propensity (Na4vSS) of the sets corresponding to each group considering only domains or whole proteins, respectively. **(C)** and **(D)** Distribution of the average aggregation propensity (Na4vSS) across the sets corresponding to the domain groups that show the most opposed behavior: Other (blue) and CAMK (red). Distributions are shown for kinase domains only **(C)** and for whole proteins **(D)**.

Typical protein kinase domains share a common catalytic core consisting of a small, mostly β-sheet, N-terminal subdomain and a larger, mostly α-helical, C-terminal subdomain (Taylor and Radzio-Andzelm, 1994). Despite sharing a common structural core, typical human protein kinases show significant sequential divergence and can be classified in 119 different families corresponding to 9 major groups (Manning et al., 2002b), excluding atypical protein kinases, which do no have structural similarity to the rest of protein kinases. The proteins in a given group are sequentially related. Therefore, we explored if the different groups have characteristic aggregative and amyloidogenic properties. For statistical purposes we analyzed only those groups containing at least 40 sequences: AGC (67 domains), CAMK (69 domains), CMGC (64 domains), Other (80 domains), STE (47 domains), Tyrosine kinase (93 domains), and Tyrosine kinase-like (41 domains). This subset accounts for 89% of human protein kinase sequences. In Figure 7 we compare the Na4vSS values for the kinase domains and the complete proteins. With the exception of those in the heterogeneous group Other, the domains in the rest of the groups display positive average aggregation propensity (Figure 7A), with CAMK and STE groups displaying the highest and lowest aggregation propensities, respectively. These differences might reflect functional constrains, since kinases in the CAMK group tend to form complexes by auto-association whereas STE kinases act transducing signals from the surface of the cell to the nucleus and should be inherently soluble. All full-length proteins display negative Na4vSS values (Figure 7B). The analysis of Na4vSS cumulative frequencies in different groups does not show any correlation between the aggregation propensity of the domain and that of the protein within it is included (Figures 7C,D).

**Figure 8:**
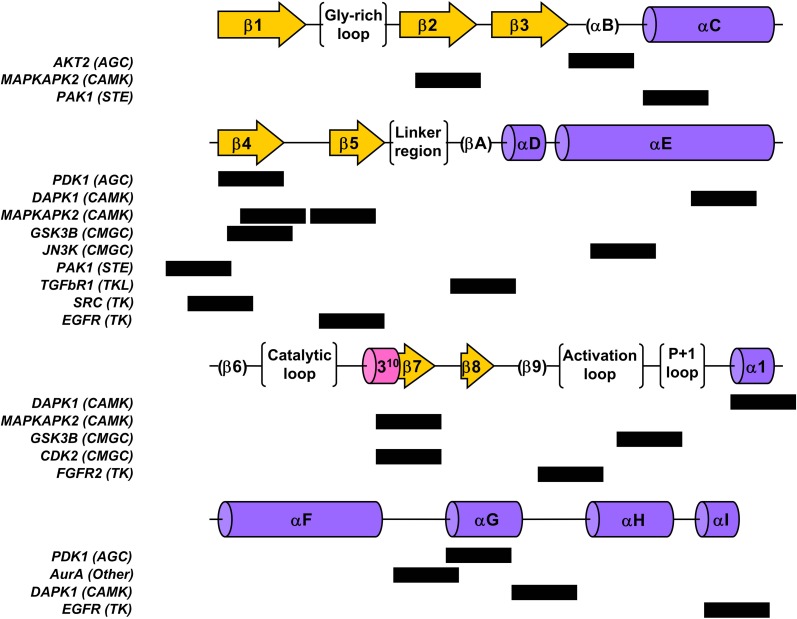
**Location of the detected amyloidogenic patterns within the global kinase fold structural elements for a representative set of the different groups of human kinase domains**. Structural elements are depicted following the nomenclature in (Scheeff and Bourne, 2005); β-sheets are shown as arrows, α-helices as cylinders and other structural or functional features are shown in parenthesis or brackets. Black boxes denote the location of the hexapeptidic pattern found for the corresponding inline domain.

**Figure 9:**
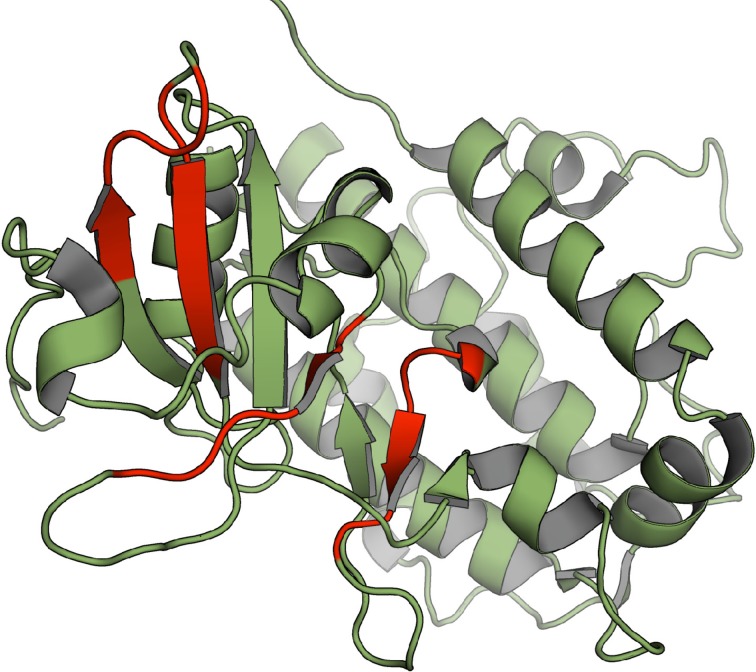
**Location of the detected amyloidogenic patterns on the structure of MAPKAPK2 belonging to the CAMK group (PDB 1kwp), amino acid stretches corresponding to the detected patterns are shown in red**.

We used the structural alignment reported by Scheeff and Bourne (Scheeff and Bourne, 2005) to map the above mentioned amyloidogenic hexa-peptides over the three-dimensional structures of kinase domains. Thirteen non-redudant structures, representative of the domains in the different human groups, were selected for the analysis (Figure 8). About 90% of the predicted amyloidogenic regions overlap with secondary structure elements in the kinase domains. These stretches are sequence dependent and, therefore, they map in different regions in the different domains; however the region comprising the well conserved β-hairpin formed by β-sheets 4 and 5 at the N-terminal subdomain seems to be specially amyloidogenic. Importantly, the hydrophobic side chains in these two β-sheets are usually buried inside the structure, thus preventing the aggregation of the native domain under physiological conditions. The amyloidogenic regions in the mitogen-activated protein kinase-activated protein kinase 2, belonging to the CAMK group, which include β-sheets 4 and 5, are shown in Figure 9 over the domain structure.

### Aggregation properties of protein kinase groups in different species

**Figure 10:**
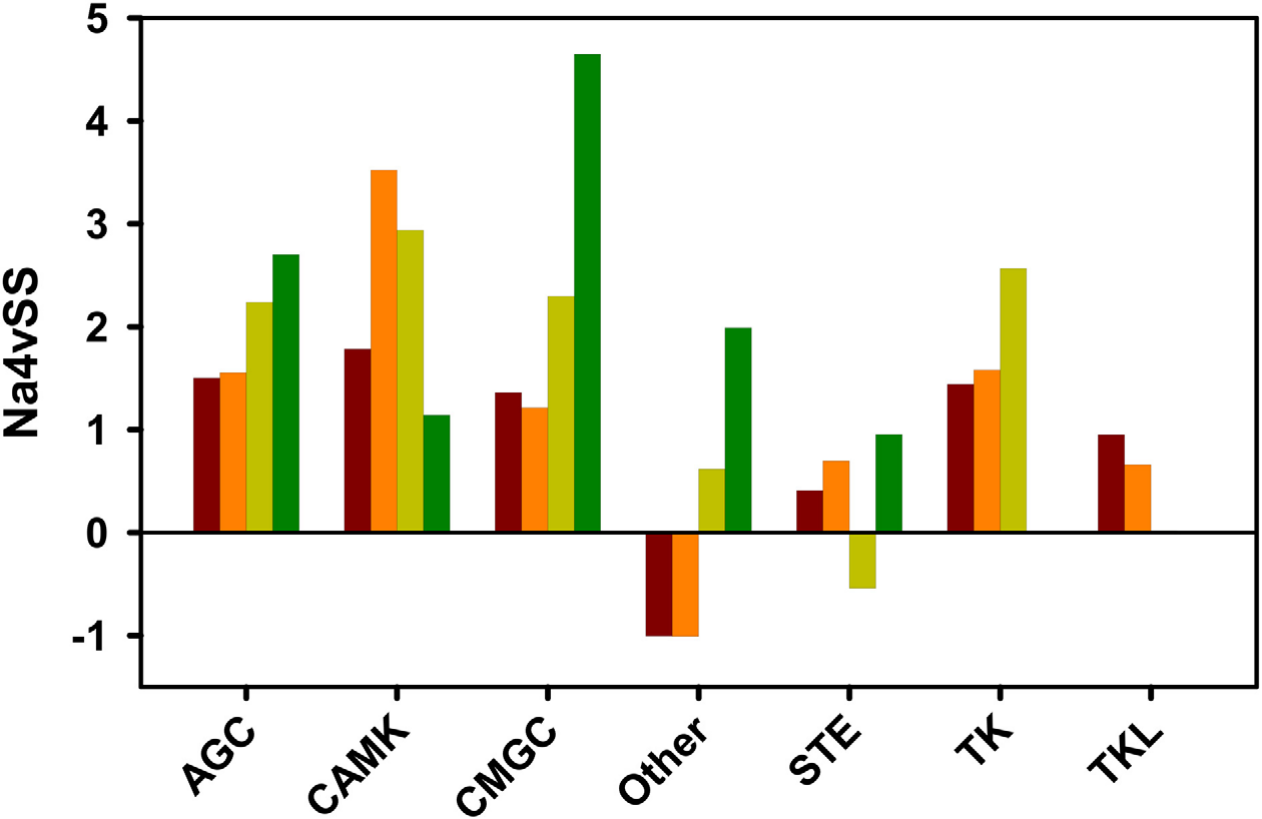
**Average aggregation propensity (Na4vSS) for discrete domain datasets of kinase groups in different organisms: human (brown), mouse (orange), fruit fly (light green), and budding yeast (dark green)**. Only the groups considered previously in the human kinome analysis are represented.

To test if the observed relationship between domains aggregation propensity and organisms complexity is maintained at the group level, we compared the Na4vSS of human kinase groups with those in yeast, fly, and mouse (Figure 10). It is important to note that the human kinome contains two and five times more kinases than the kinomes of fly and yeast, respectively. Therefore, the number of sequences in the considered groups differs between organisms. A strict correlation between aggregation propensity and organism complexity is seen for domains belonging to kinases in the AGC, CMG, Other, and TK Tyrosine kinase groups. However, deviation from this principle is observed in the CAMK group in which yeast domains appear to be less aggregation-prone than those in multicellular organisms. This might reflect the fact that three out of the seven yeast-specific kinase subfamilies and 30% of the specific yeast kinase genes belong to the CAMK group (Manning et al., 2002a). They mediate essentially unicellular-specific functions (Ball et al., 2000), including osmotic and other stress responses, which require a significant degree of solubility.

**Figure 11:**
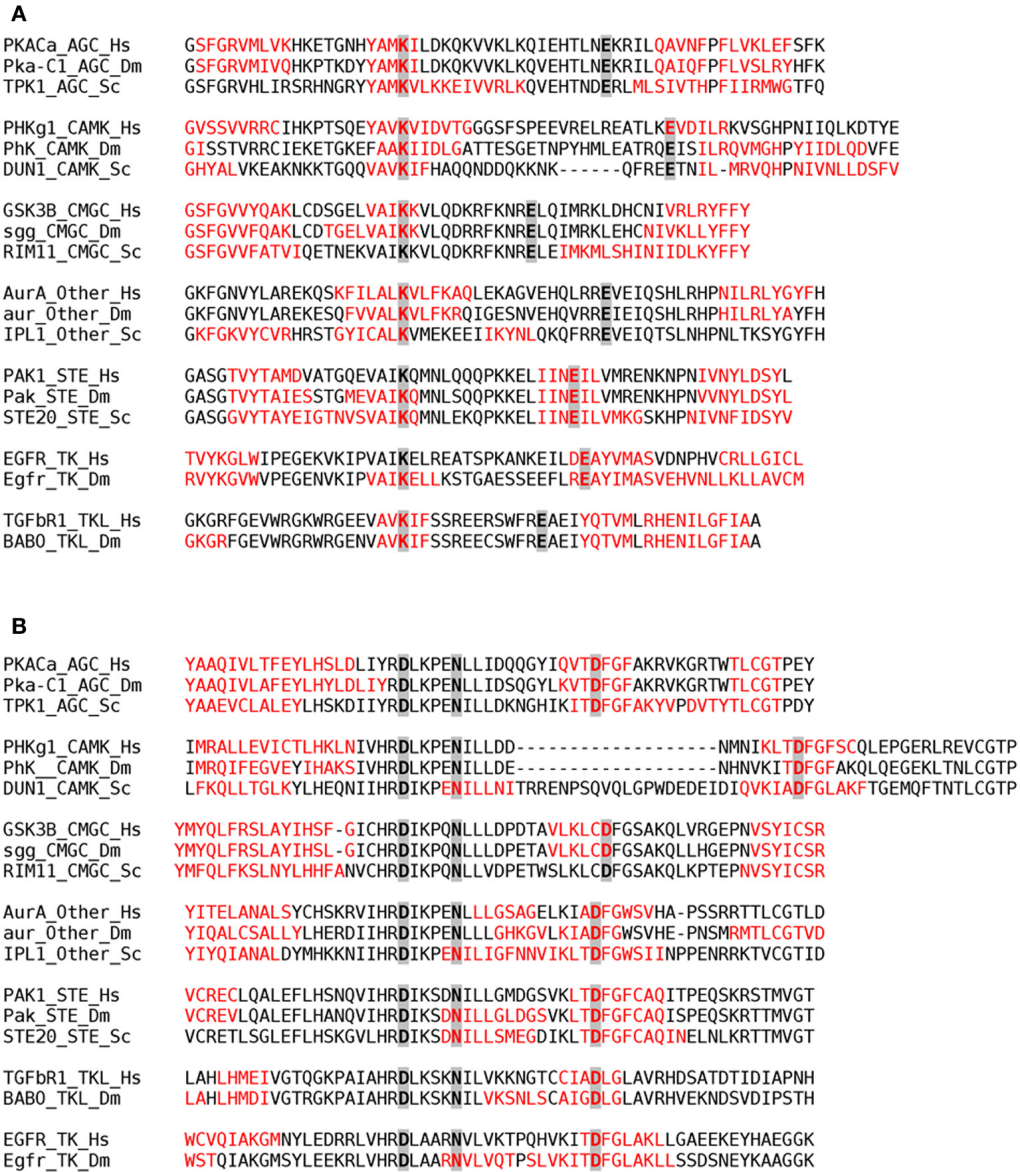
**Aggregation-prone regions detected by AGGRESCAN within the 20 amino acids flanking the Nter and Cter sides of the first (A) and second (B) regions containing conserved catalytic residues in kinase domains**. Aggregation-prone stretches are shown in red in the alignment of representative proteins corresponding to different classes (AGC, CAMK, CMGC, Other, STE, TK, and TKL) in human (Hs), fruit fly (Dm), and yeast (Sc). The conserved catalytic residues are highlighted in gray.

We addressed whether the location of catalytically important residues might constraint the evolution of aggregation propensity. To this aim, we analyzed the presence of conserved catalytic residues inside aggregation-prone stretches in representative proteins corresponding to different groups in human, fly, and yeast (Figure 11). The results indicate that, independently of the protein group and species, a significant amount of catalytic residues are embedded in aggregation-prone regions, likely limiting the evolution of these regions toward more soluble sequences.

## Discussion

Computational predictions of the aggregation propensities of proteomes in different organisms suggest that minimization of protein aggregation may act as an important evolutionary constrain, shaping proteins and cellular machineries (Castillo et al., 2011). This negative selection pressure has been suggested to modulate the aggregation propensity of protein sequences according to their biological context (Rousseau et al., 2006b; Tartaglia et al., 2007; Monsellier et al., 2008; Castillo and Ventura, 2009; Tartaglia and Vendruscolo, 2009; de Groot and Ventura, 2010). In this line, a previous analysis of the overall aggregation tendency of complete proteomes, including those of yeast, fly, mouse, and human, has shown that this value decreases with increasing organism complexity and longevity (Tartaglia et al., 2005). This trend results from the fact that higher organisms contain fewer sequences with high aggregation propensity, but specially because their proteomes contain a higher proportion of IUPs. In addition, the force of natural selection against the aggregation of a given protein depends on the selective contribution of this protein to the organism fitness (Chen and Dokholyan, 2008; Villar-Pique et al., 2012). Therefore, despite the analysis of complete proteomes has contributed significantly to our present understanding on how aggregation modulates protein sequences and structures, the datasets used in these studies comprise proteins displaying divergent structures, from globular to unfolded, and functions, from essential to spurious, and thus under different evolutionary pressures.

Most evolutionary studies rely on the analysis of sequential or structural differences in a given protein family or super-family. By analogy, the study of the aggregation properties of a homogeneous, but relatively large, group of proteins sharing the same fold and function in different organisms might help to confirm the constraint aggregation exerts in protein evolution. The cellular role of the proteins in this set should be important enough to be the subject of a significant selection against their aggregation, in order to avoid a deleterious loss of function. The superfamily of protein kinases is perfectly suited for this purpose. We could confirm here that, on the average, the kinase domains of more complex and longer living organisms tend to have lower aggregation propensities and less potent aggregating peaks in their sequence.

Our predictions argue that, independently of the considered organism and group, kinase domains have significant aggregation tendency. Accordingly, human domains display higher aggregation propensity than the selected ensemble of folded proteins. Interestingly enough, it has been recently shown that 60% of human kinases are clients of the HSP90 chaperone and that their binding determinants are located in the kinase domain (Taipale et al., 2012). In these cases, inhibition of the chaperone binding activity results in dissociation of the kinase domain and leads to aggregation, supporting the view that kinase domains are intrinsically aggregation-prone. This property might explain why, when recombinantly expressed in bacteria, kinase domains accumulate as misfolded and insoluble aggregates in many cases (Benetti et al., 1998; Marin et al., 2010) since prokaryotic HSP90 does not chaperone kinases (Buchner, 2010).

Despite the recognized cytoxicity of amyloid aggregates, it is now clear that amyloidogenic sequences are ubiquitous in all the proteomes (Castillo et al., 2011), supporting the view that most polypeptides share the potential to form amyloid-assemblies (Dobson, 2004). Accordingly, we found that 85% of the human kinase domains include at least one amyloidogenic stretch and in many cases several of them. These data are in agreement with the observation that, *in vitro*, the population of partially unfolded states results in amyloid aggregation in different, unrelated, kinase domains (Damaschun et al., 2000; Agocs et al., 2010, 2012; Georgescauld et al., 2011). The mapping of these amyloidogenic regions on the three-dimensional structures of catalytic domains belonging to different human kinase groups indicates that, in most cases, they overlap with regular elements of secondary structure, providing support to the hypothesis that the molecular determinants responsible for amyloid formation coincide with those maintaining the native structure of proteins (Sabate et al., 2012). In other words, kinase domains cannot avoid the presence of amyloid regions simply because they need them to fold into compact and stable globular conformations. Moreover, aggregation-prone regions overlap with conserved functional residues, suggesting that the selection for active conformations during evolution constrains the pressure to attain more soluble sequences.

In the native state, the side-chains of amyloidogenic sequences are essentially protected from the solvent inside the three-dimensional structure of kinase domains. However, mutations or environmental conditions that destabilize the functional conformation would result in their partial or total exposition to solvent, allowing them to nucleate amyloid self-assembly, a mechanism common to several proteins involved in human conformational disorders (Monsellier and Chiti, 2007). This model would explain why certain kinases, such as c-Src and EGFR, associate transiently with HSP90 chaperone during maturation, where these regions are likely exposed to solvent, but do not bind to this chaperone when they are fully folded (Xu et al., 1999, 2005).

Despite the effect of selective pressure could be clearly traced for kinase domains, full-length kinases display, on the average, low aggregation propensity, independently of the considered species. Our analysis indicates that the presence of intrinsically unstructured regions in non-catalytic domains is, at least in part, responsible for the low sequential aggregation propensity of kinases. It has been recently shown that intrinsically disordered protein sequences traslationally fused to globular proteins act as entropic bristles, providing them solubility by creating both a large favorable surface area for water interactions and large excluded volumes around the partner (Santner et al., 2012). In kinases, this effect seems likely to act as a compensatory mechanism for the obligatory presence of aggregation-prone sequences in the catalytic domain.

Apart from structural and catalytic constrains, there is an alternative and, although speculative, more interesting explanation for the calculated aggregation propensity of kinase domains: that it serves for functional purposes. It has been shown that despite kinases can rapidly evolve away from chaperone assisted folding they have generally not done so. This suggests that kinases might specifically exploit association with the chaperone machinery as a means of regulation, in such a way that the inherent instability of some kinases might in fact be employed as a mechanism to provide fidelity in regulatory cascades (Taipale et al., 2012). The exposition of pre-existing aggregation-prone regions able to bind the chaperone upon partial unfolding would likely play an important role in this mechanism.

Despite the present work illustrates the utility of prediction algorithms to provide insights on the relationship between protein aggregation and sequence evolution, it should be noted that at the present moment these algorithms do not allow to evaluate the impact of post-translational modifications on aggregation propensity. It is clear that incorporating these modifications, which might promote extensive structural rearrangements, in the calculations would result in a more realistic readout on the evolutive constrains imposed by protein aggregation and that we should work toward this objective.

## Materials and methods

### Dataset curation

Kinase Domains and Entire Protein sequences where retrieved from the Kinbase Database (http://kinase.com/kinbase/) for *Homo sapiens*, *Mus musculus*, *Drosophila melanogaster*, and *Saccharomyces cerevisiae*. Domains or proteins with “not available” (“N/A”) sequence or containing the non-amino acidic character “^*^” were deleted. Whenever the non-amino acidic character “X” was found, it was substituted by alanine (“A”), unless more than three consecutive “X” were found, in which case the sequence was deleted. Single spaces found within sequences were suppressed.

Sequences in Kinase Domain datasets were verified to correspond to sequential regions of the Entire Protein datasets. Those domains without a correspondent sequence in the Entire Protein dataset were deleted. In the same way, full-length proteins without an associated kinase domain were also suppressed.

The Kinase Domain and Entire Protein datasets obtained in this way were further subdivided according to the different groups of kinases present in each species.

Due to AGGRESCAN technical limitations, parameters for sequences larger than 2000 amino acids could not be computed and those sequences and their corresponding domains were deleted from the datasets.

### AGGRESCAN calculations

AGGRESCAN (http://bioinf.uab.es/aggrescan/) was employed, with default settings, in order to compute parameters indicative of aggregation properties for every single sequence, as well as overall values for complete datasets. In order to be able to compare the large datasets of this study, only normalized values were considered (Figure 1). For further information about the calculation of AGGRESCAN parameters and its applications the reader is referred to AGGRESCAN Help File (accessible from the server front page) and to the published tutorials (de Groot et al., 2012).

### Composition of kinase domains

The frequencies of occurrence of each amino acid were calculated for the complete kinase domains datasets of human and budding yeast. In order to look for compositional biases in these sets, frequency differences were calculated relative to the occurrence of each amino acid in the complete Swiss-Prot database release 2012_07.

### Analysis of the human kinome

In order to be able to set up general comparisons between the human kinase proteins and its isolated domains, a reference dataset was defined by randomly retrieving, using a randomizing function, 500 sequences from the subset of sequences with less than 40% identity of the ASTRAL Compendium (Astral40), whose AGGRESCAN parameters were also calculated.

### Detection of amyloidogenic patterns

The human kinase domains dataset was explored to identify amyloidogenic patterns by using the amyloidogenic signature {P}-{PKRHW}-[VLSWFNQ]-[ILTYWFN]-[FIY]-{PKRH} defined in (de la Paz and Serrano, 2004) using the ScanProsite web server.

### Intrinsical unfolding prediction

The intrinsical unfolding potential of selected subsets of the human and yeast kinase domains datasets was computed using the FoldIndex webserver with default settings.

### Conflict of interest statement

The authors declare that the research was conducted in the absence of any commercial or financial relationships that could be construed as a potential conflict of interest.

## Research Topic:

From structural to molecular systems biology: experimental and computational approaches to unravel mechanisms of kinase activity regulation in cancer and neurodegeneration

## Topic Editor:

Matteo Barberis, Humboldt University Berlin, Germany; Max Planck Institute for Molecular Genetics, Berlin, Germany
